# Supplementary material for: PgRsp Is a Novel Redox-Sensing Transcription Regulator Essential for Porphyromonas gingivalis Virulence
Source: Microorganisms. 2019 Nov 28;7(12):623. doi: 10.3390/microorganisms7120623 (PMC6955866; doi:10.3390/microorganisms7120623)
Supplement: Supplementary file 1 [file microorganisms-07-00623-s001.pdf]

Table S1. Primers used in this study

Primer name	DNA sequence	Gene	Description	References
F1_PG1573_mut	5′GGAGAACTCCGACGGGTG 3′	*PGA7_00004090* (*pgrsp*)	Amplify DNA fragments; including sequences flanking the *pgrsp* gene, as well as *ermF* cassette for construction of TO11 mutant strain.	This study
R1_PG1573_mut	5′CGCATAACGGCTGGCCACAAGCTCAGGCGAAGAG 3′			
F3_PG1573_mut	5′GAGACAACCGCGGTGCCGAACTCGTGGAGGGTA 3′			
R3_PG1573_mut	5′TGCCGATAGCTTGTTTCATCG 3′			
F2_PG1573_mut	5′CTTCGCCTGAGCTTGTGGCCAGCCGTTATGCGGCAG 3′	*ermF*		
R2_PG1573_mut	5′CCTCCACGAGTTCGGCACCGCGGTTGTCTCTCTTTC 3′			
F_PG1573_cmpl	5′GATCCTCGAGCTTCCAATACACGGGGAAATTCAC 3′	*PGA7_00004090* (*pgrsp*)	Amplify DNA fragment of *pgrsp* gene, including promoter sequence. Used for cloning into pTIO-tetQ plasmid.	This study
R_PG1573_cmpl	5′GATCGGATCCTTACATAAGGGTGATTTTGTTCGCC 3′			
F_PG1573_MBP	5′CTCGGGATCGAGGGAAGGATGAAGCAGATAGAAGATATATCCG 3′	*PGA7_00004090* (*pgrsp*)	Amplify DNA fragment of *pgrsp* gene used for cloning into pMAL_c5x_His plasmid.	This study
R_PG1573_MBP	5′CCTGCAGGGAATTCGGATCCTCACATAAGGGTGATTTTGTTACG 3′			
F_PgRsp_C76A	5′ACTATATCAAGGCAGGCGAAAGCGCTATCATGTCTTTTCTTGGG 3′	*pgrsp C76A*	Primers used to introduce point mutations in the sequence encoding PgRsp protein, cloned into pMAL_c5x_His plasmid.	This study
R_PgRsp_C76A	5′CCCAAGAAAAGACATGATAGCGCTTTCGCCTGCCTTGATATAGT 3′			
F_PgRsp_M78A	5′AAGGCAGGCGAAAGCTGTATCGCGTCTTTTCTTGGGGGCCTGCAC 3′	*pgrsp M78A*		
R_PgRsp_M78A	5′GTGCAGGCCCCCAAGAAAAGACGCGATACAGCTTTCGCCTGCCTT 3′			
F_PgRsp_H85A	5′CTTTTCTTGGGGGCCTGGCCAATGAGACAAGCAAGGTGAAGG 3′	*pgrsp H85A*		
R_PgRsp_H85A	5′CCTTCACCTTGCTTGTCTCATTGGCCAGGCCCCCAAGAAAAG 3′			
rtFUR6F	5′TTCTGCGTTTGCCTTCTCCC 3′	*PGA7_00014570* (*pgfur*)	Amplify DNA fragment of the gene in RT-qPCR.	[13]
rtFUR6R	5′TGAGATCCTTGTCGGCCAGT 3′			
16SrRNA-F	5′GCTTCGAAATACGAAACGTG 3′	*PGA7_00000960* (*16S rRNA*)	Amplify DNA fragment of the gene in RT-qPCR.	[S1]
16SrRNA-R	5′TATATCCGTCTGTCGGAACG 3′			
HYq4_F	5′CTTGACTTCAGTGGCGGCAG 3′	*PGA7_00004270* (*hmuY*)		
HYq4_R	5′AGGGAAGACGGTTTTCACCA 3′			
F_PG1573	5′TTTGAATGAGGGCCAGTAGG 3′	*PGA7_00004090* (*pgrsp*)	Amplify DNA fragment of the gene in RT-qPCR.	[16]
R_PG1573	5′AAGGTGGAGATCGAAGACGA 3′			
F_PG0270	5′GTTGGACGAAGGTCATTGCT 3′	*PGA7_00002460* (*oxyR*)		
R_PG0270	5′TGCTCTACGGTCAGTTGTGG 3′			
F_PG0506	5′AATGATAAGCCTTATACTGTAGCTG 3′	*PGA7_00014250* (*rgpB*)		
R_PG0506	5′GTTTTGTGCTTCGAATACCATGC 3′			
F_PG0553	5′GAGGGACTTTGCCTGACATC 3′	*PGA7_00013810*		
R_PG0553	5′GCACCACACCGTTCTTCTCT 3′			
F_PG2024	5′GGACAAGGACCGACGAAAG 3′	*PGA7_00018890* (*rgpA*)		
R_PG2024	5′TATAGGTGTAATCGCTTCCACC 3′			

F_PG1844	5′GAGTGTGGGTGCTAATGCCG 3′	*PGA7_00017110*		
R_PG1844	5′CACCAATATGGGTAATATTGCCG 3′	(*kgp*)		
F_sod	5′GCCGAATTGTTTGTCGATAG 3′	*PGA7_00004330*	Amplify DNA fragment of the gene in RT-qPCR.	This study
R_sod	5′AATTCCACCACGGTAAGCACC 3′	(*sod*)		
F_fimA	5′CTATCCTGGTGGGACTGCATC 3′	PGA7_00019920		
R_fimA	5′ACCAAAGAATTGCCGAAAATC 3′	(*fimA*)		
F_bcp	5′TGAGACGCACTTCCTCATCC 3′	*PGA7_00010740*		
R_bcp	5′TATCGCGGACGAAAGATCGC 3′	(*bcp*)		
F_ ahpC	5′CTACGCTGTTGTCGTTCAGC 3′	*PGA7_00013260*		
R_ahpC	5′TTGCGATACCCACTTCGTAC 3′	(*ahpC*)		
F_dps	5′GAGCGTATCCTCCAACTTGG 3′	*PGA7_00000850*		
R_dps	5′AATCCACCGTTACCTCATCG 3′	(*pgdps*)		
F_ tpx	5′CGCCGTTTCAATCAGGAAGC 3′	*PGA7_00002640*		
R_ tpx	5′GCCAAGAGACCTTTCAAAGGG 3′	(*tpx*)		
F_ rbr	5′GCCGTATTTCGTCAGATAGC 3′	*PGA7_00001850*		
R_ rbr	5′TTCATTGGCTCGAAGTAAGCC 3′	(*rbr*)		
F_primer	5′ATGCAACGCATTGCCG 3′	*PGA7_00002300*	Amplify DNA fragment of the gene in RT-qPCR.	[S2]
R_primer	5′TGCATTGGCTTCGCC 3′	*(ustA)*		
F_hmu_EMSA	5′CGGAATAATCGGCTGATACAC 3′	*PGA7_00004270*	Amplify DNA fragments of the genes used as probes in EMSA; forward primers were used with or without biotin attached to the 5′ end.	[16]
R_hmu_EMSA	5′TAGAGACACAATCAATGGCAATG 3′	(*hmuY*)		
F_bcp_EMSA	5′TCATGAGCTTTAGATGTACGTTAG 3′	*PGA7_00010740*	Amplify DNA fragments of the genes used as probes in EMSA; forward primers were used with or without biotin attached to the 5′ end.	This study
R_bcp_EMSA	5′TCAGGAATACGGTCTCCTATTTGT 3′	(*bcp*)		
F_PgRsp_EMSA	5′GACTGCTGTGCTTATACTTGAAAAAC 3′	*PGA7_00004090*		
R_PgRsp_EMSA	5′AGGATGCCATGCTTATACAGTTTG 3′	(*pgrsp*)		

S1. Gmiterek, A., Wójtowicz, H., Mackiewicz, P., Radwan-Oczko, M., Kantorowicz, M., Chomyszyn-Gajewska, M., Frąszczak, M., Bielecki, M., Olczak, M., Olczak, T. The unique hmuY gene sequence as a specific marker of *Porphyromonas gingivalis*. *PLoS One*. **2013** 2;*8(7)*:e67719. doi: 10.1371/journal.pone.0067719.

S2. Boutrin, M.C., Wang, C., Aruni, W., Li, X., Fletcher, H.M. Nitric oxide stress resistance in *Porphyromonas gingivalis* is mediated by a putative hydroxylamine reductase. *J Bacteriol*. **2012** *194(6)*:1582-92. doi: 10.1128/JB.06457-11.
